# Effect of HIV on mortality among hospitalised patients in South Africa

**DOI:** 10.4102/sajhivmed.v24i1.1477

**Published:** 2023-04-26

**Authors:** Dirk J. Lamprecht, Neil Martinson, Ebrahim Variava

**Affiliations:** 1 Department of Internal Medicine, Faculty of Health Sciences, University of the Witwatersrand, Johannesburg, South Africa; 2 Perinatal HIV Research Unit, University of the Witwatersrand, Johannesburg, South Africa; 3 Johns Hopkins University Center for TB Research, Johns Hopkins University, Baltimore, United States of America; 4 Department of Internal Medicine, Klerksdorp/Tshepong Hospital Complex, Faculty of Health Sciences, University of the Witwatersrand, Johannesburg, South Africa

**Keywords:** in-hospital mortality, cause of death, HIV, mortality rate, people living with HIV, tuberculosis-related mortality

## Abstract

**Background:**

HIV and AIDS continues to impose substantial healthcare challenges in sub-Saharan Africa, but there are limited local data comparing inpatient outcomes between people with HIV (PLWH) and those uninfected.

**Objectives:**

To compare cause-specific mortality among hospitalised adolescents and adults, stratified by HIV-serostatus.

**Method:**

A cross-sectional analysis was performed, analysing cause-specific inpatient mortality data and total admissions, from 01 January 2017 to 30 June 2020, at Tshepong Hospital, North West province, South Africa.

**Results:**

The overall inpatient mortality rate decreased from 14.5% (95% confidence interval [CI]: 13.4–16.0) in 2017, to 11.3% (95% CI: 10.6–11.9) in 2020; *P* < 0.001. People living with HIV accounted for 53.9% (*n* = 2342) of inpatient deaths, 22.6% (*n* = 984) were HIV-seronegative patients and 23.5% (*n* = 1020) patients with unknown HIV-serostatus. People with HIV died at younger ages (median: 44 years, interquartile range [IQR]: 35.8–54.2) compared to HIV-seronegative inpatients (median: 64.4 years, IQR: 55.5–73.9); *P* < 0.001. Leading causes of death were pneumonia (19.9%, *n* = 863), then pulmonary and extrapulmonary tuberculosis (15.0%, *n* = 654). People with HIV who had CD4+ counts < 350 cells/mL or viral load ≥ 1000 copies/mL had increased risk of death from tuberculosis compared to virally suppressed patients (adjusted relative risk: 2.10 [95% CI: 1.44–3.04, *P* < 0.009] and 1.56 [95% CI: 1.22–2.00, *P* < 0.001]).

**Conclusion:**

Our study, conducted in a regional hospital in South Africa, showed PLWH had higher mortality rates and died at younger ages compared to HIV-seronegative patients.

**What this study adds:** Our study adds key mortality data, including causes of death in, by HIV status. While antiretroviral treatment decreases mortality, the management of communicable diseases requires further improvement.

## Background

HIV and AIDS impose substantial economic and healthcare challenges, especially in sub-Saharan Africa.^1,2,3^ Death ascribed to HIV and AIDS-related illnesses decreased markedly after the introduction, and subsequent widespread availability, of antiretroviral treatment (ART).^1,4,5,6,7^ Despite this, the prevalence of HIV infection in South Africa in 2022 was 13.9%, equating to 8.45 million people living with HIV (PLWH).^8^

Changing patterns have emerged regarding cause-specific mortality in PLWH, both globally and in South Africa.^9,10,11,12^ Upscaling of ART has led to increased survival among PLWH, decreased mortality related to communicable diseases, and a decreasing overall crude death rate.^8,13^ Despite this, sub-Saharan Africa remains burdened with high in-hospital mortality rates amongst PLWH.^14^ Several studies have reported reduced inpatient mortality rates in PLWH receiving ART.^12,15,16,17^ However, PLWH continue to have higher mortality rates than HIV-seronegative inpatients.^12,15,16,17^

Understanding changing mortality patterns is important in terms of policy and budgeting. However, there are insufficient local data comparing outcomes and burden of disease amongst PLWH and HIV-seronegative inpatients. This study describes inpatient mortality rates, at a regional healthcare facility in South Africa, over a period of 42 months. We compared the differences in cause-specific mortality between adolescent and adult PLWH and HIV-seronegative inpatients.

## Methods

A cross-sectional analysis was conducted at a regional hospital in the North West province of South Africa. We analysed routinely collected data, describing mortality among hospitalised adolescents and adults between 01 January 2017 and 30 June 2020. Patients ≥ 15 years of age admitted to adult wards at Tshepong Hospital were considered eligible for inclusion in the study.

Two routinely collected databases were used in these analyses. The first included the number of admissions to medical wards each calendar month, stratified by year of admission, gender, and HIV-serostatus. There were no individual admission or discharge diagnoses, nor outcome data available to discriminate between patients who survived or died.

The second database was generated by weekly, standardised mortality meetings. The meetings were held throughout the study period and headed by at least two senior physicians. The same senior physicians supervised the process, ensuring a standardised approach of investigation and classification. To accurately complete statutory death notification forms, medical records and laboratory and histological results were discussed. The *immediate, underlying* and *contributory* causes of death were determined, and the outcome data for each case was entered into this second database (paper-based and electronic).

Additional variables entered into this mortality database included: demographic information, dates of admission and death, HIV-serostatus, ART history (including ART non-adherence), tuberculosis co-infection, haemoglobin concentration, CD4+ counts, and viral load (VL). Laboratory results obtained during admission were rechecked at the mortality meetings, and afterwards rechecked using the National Health Laboratory Service electronic database before being recorded in the local database. The database was deidentified prior to analysis; names were replaced by initials, and dates of birth were converted to ages at death, prior to analysis. The causes of death were categorised according to the International Statistical Classification of Diseases, Tenth Edition (ICD-10), mortality coding system.^18^ The initial paper-based and electronic database a were compared and rechecked to ensure imputation errors were not made and that missing or incorrect data were kept at a minimum. If records were incomplete and causes of death not reliably determined, the specific cases were used in the calculation of overall mortality rates, but not included in the evaluation of cause-specific mortality.

Overall mortality rates were determined by using the total admission numbers as the denominator. Similarly, mortality rates in PLWH were determined by using the number of HIV-seropositive admissions as the denominator. Due to the unclear distribution of denominator data for patients without HIV (HIV-seronegative and unknown HIV-serostatus), these two groups were combined to calculate mortality rates.

## Data analysis

The data set was imported to STATA^®^ 14.2 (College Station, Texas, United States for analysis. Descriptive statistics were used to describe the inpatients who died during the period of interest. Percentages and frequencies were used to describe the categorical data. For the description of continuous variables, medians and interquartile ranges (IQR) were used. Mortality rates were determined as the total number of inpatient deaths divided by total admissions for the same period and expressed as percentages with a 95% confidence interval (CI). The chi-squared test was employed, to test for differences in mortality rate trends, between groups stratified by HIV-serostatus.

The leading causes of death overall were explored, as well as for groups stratified by HIV-serostatus, age categories, most recent CD4+ counts, and VL results within 6 months of death. We defined patients on ART with VL ≤ 50 copies/mL (within 6 months preceding death) as being virally suppressed.^19^ Proportions of the leading (*immediate, underlying* and *contributory*) causes of death were determined, and compared across categories.

Univariable and multivariable Poisson regressions with robust error variance were used to determine the factors associated with the leading causes of death: pneumonia and tuberculosis (pulmonary and extrapulmonary). Viral, bacterial (excluding mycobacterium tuberculosis) and fungal infections of the lower respiratory tract were classified as pneumonia. Age, gender, year of death, HIV-serostatus, as well as most recent CD4+ counts and VL results, were included in a priori multivariable models, and we reported adjusted relative risks (aRR). *P*-values of less than 0.05 were determined to represent statistically significant differences.

## Results

**FIGURE 1:**
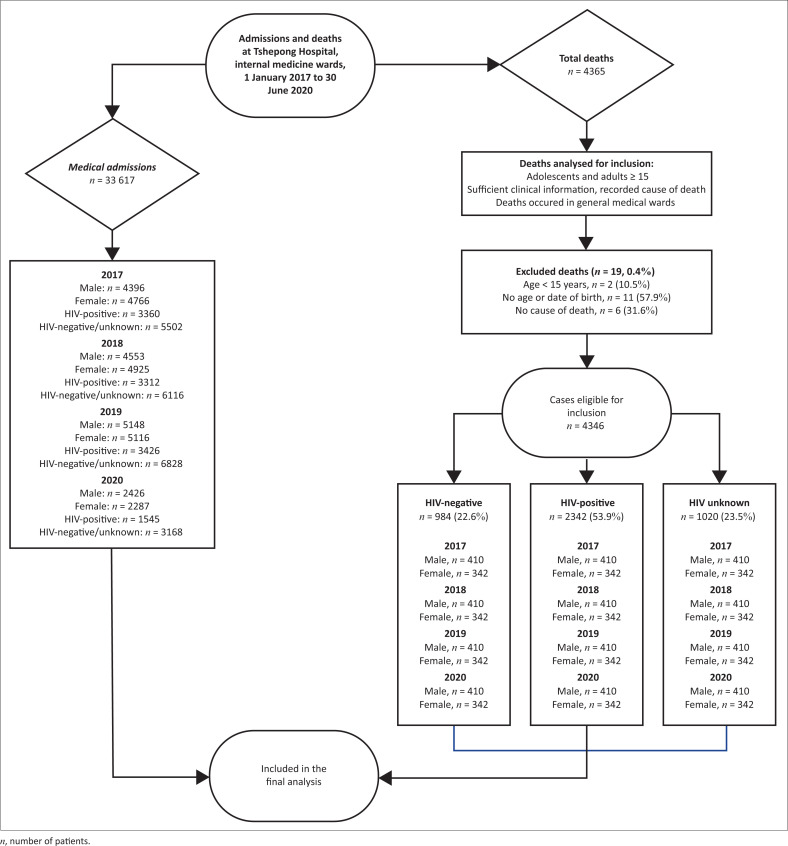
All patients admitted to, and inpatient deaths that occurred at, the medical wards of Tshepong Hospital, from 01 January 2017 to 30 June 2020. The phases of the study, as well as included and excluded patients are depicted.

We reviewed 33 617 adult admissions, to the internal medicine inpatient service, during the study period (Figure 1, Online Appendix 1 Table 1). Similar numbers of women (50.9%, *n* = 17 094) and men (49.1%, *n* = 16 523) were admitted. However, more in-hospital deaths occurred in men (53.5%, *n* = 2323) than in women (46.5%, *n* = 2023); *P* = 0.404. Of the total admissions, 35.6% (*n* = 11 953) were PLWH, with the remainder being either HIV-seronegative or having unknown HIV-serostatus (64.4%, *n* = 21 664). A total of 4346 inpatient deaths occurred during the study period; most (53.9%, *n* = 2342, *P* < 0.001) were in PLWH, 22.6% (*n* = 984) were HIV-seronegative, and 23.5% (*n* = 1020) had unknown HIV-serostatus at the time of death.

Overall, the median age at death was 53 years (IQR: 40.6–67.2). People living with HIV died at a median age of 44 years (IQR: 35.8–54.2), HIV-seronegative patients at 64.4 (IQR: 55.5–73.9), and those with unknown HIV-serostatus at 68.4 (IQR: 58.4–77.3); *P* ≤ 0.001. The median duration from admission until death was 4 days overall (IQR: 1–9). The median duration of admission until death was 4 days (IQR: 2–9) for PLWH, 6 days (IQR: 2–11) for HIV-seronegative patients, and 2 days (IQR: 1–5) for those with unknown HIV-serostatus; *P* ≤ 0.001.

The median haemoglobin concentration, in those who died, was 10 g/dL (IQR: 8.5–13.1) overall, 8.9 g/dL (IQR: 6.9–11.2) in PLWH, 10.8 g/dL (IQR: 8.5–13.1) in HIV-seronegative inpatients and 12.1 g/dL (IQR: 9.7–14.1) in patients with unknown HIV-serostatus. Prior tuberculosis infections were reported in 20.3% (*n* = 880) of those who died. Previous tuberculosis infections were more frequent in PLWH (31.4%, *n* = 736, *P* < 0.001), compared to HIV-seronegative patients (9.4%, *n* = 92) and those with unknown HIV-serostatus (5.1%, *n* = 52). Fifteen per cent (*n* = 654) of in-hospital deaths were caused by pulmonary or extrapulmonary tuberculosis. The highest number of tuberculosis-related deaths was observed in PLWH (24.9%, *n* = 584, *P* < 0.001). In contrast, tuberculosis was the *immediate* cause of death in only 5.4% (*n* = 53) of HIV-seronegative inpatients and 1.7% (*n* = 17) of those with unknown HIV-serostatus.

In PLWH, 60.7% (*n* = 1421) were receiving ART at time of death, 16.7% (*n* = 392) had a history of non-adherence to ART, and 17.4% (*n* = 408) died prior to ART initiation. In those who received ART, the reported regimens included non-nucleoside reverse-transcriptase-inhibitor-based (65.2%, *n* = 927) and protease-inhibitor-based treatment (12.7%, *n* = 180), while the ART regimen was unknown in 22.1% (*n* = 314). Recent VL results (within 6 months of death) were available in 69.3% (*n* = 1623) of PLWH. Of those with available VL results, 21.4% (*n* = 501) were virally suppressed, 6.9% (*n* = 162) had a VL of 51 copies/mL – 399 copies/mL, 2.1% (*n* = 48) had a VL of 400 copies/mL – 999 copies/mL, and 38.9% (*n* = 912) had a VL of ≥ 1000 copies/mL. A large proportion of patients (30.7%, *n* = 719) had no VL available prior to death. Of the PLWH that died, 59.7% (*n* = 1515) had CD4+ counts ≤ 200 cells/mL, 18.5% (*n* = 432) had CD4+ counts of 201 cells/mL – 499 cells/mL, 8.1% (*n* = 190) had CD4+ counts ≥ 500 cells/mL, and 8.8% (*n* = 205) had no results available.

**FIGURE 2:**
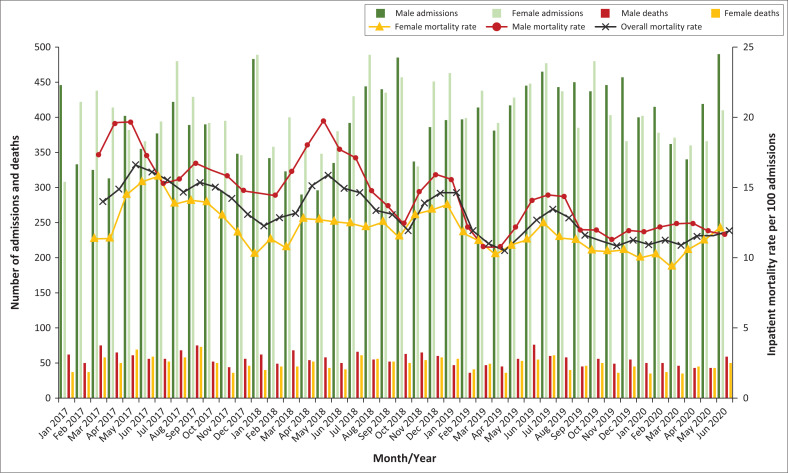
Total number of admissions and inpatient deaths at Tshepong Hospital, from 01 January 2017 to 30 June 2020. The mortality rates were determined as the number of deaths per 100 admissions and depicted as a 3-monthly moving average.

**FIGURE 3:**
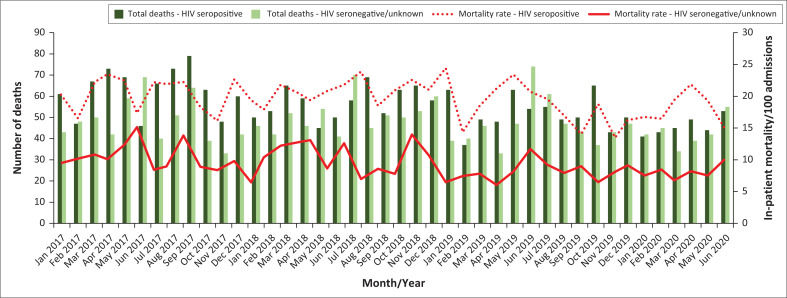
The total number of deaths for HIV-seropositive inpatients and the monthly mortality rate for people with HIV are depicted. The number of deaths and mortality rates for hospitalised patients with either a confirmed HIV-seronegative status or unknown status are also depicted for comparison. As the distribution of admissions for HIV-seronegative patients and those that had unknown HIV-serostatus were unknown, those groups were combined to calculate a mortality rate.

The inpatient mortality rate decreased from 14.5 per 100 admissions (95% CI: 13.4–16.0) in 2017, to 11.3 (95% CI: 10.6–11.9, *P* < 0.001) in 2020 (Figure 2, Online Appendix 1 Figure 1). Among men, the mortality rate decreased from 16.2 (95% CI: 14.9–18.1) in 2017, to 11.9 (95% CI: 11.3–12.7, *P* < 0.001) in 2020 (Online Appendix 1 Figure 2). The female mortality rate also decreased from 13.1 (95% CI: 12.2–14.1) in 2017, to 10.7 (95% CI: 9.6–12.1, *P* = 0.016) in 2020. Inpatient mortality rates for 2017 were higher in PLWH (20.5 [95% CI: 19.3–21.8]), than in the combined HIV-seronegative and unknown HIV-serostatus group (10.5 [95% CI: 9.9–11.5]); *P* < 0.001. Outcomes for both groups improved over time, with mortality rates of 17.8 (95% CI: 16.0–19.8, *P* = 0.011) and 8.1 (95% CI: 7.3–8.9, *P* < 0.001) per 100 admissions in 2020, for PLWH and HIV-seronegative/unknown groups respectively (Figure 3, Online Appendix 1 Figure 3).

**FIGURE 4:**
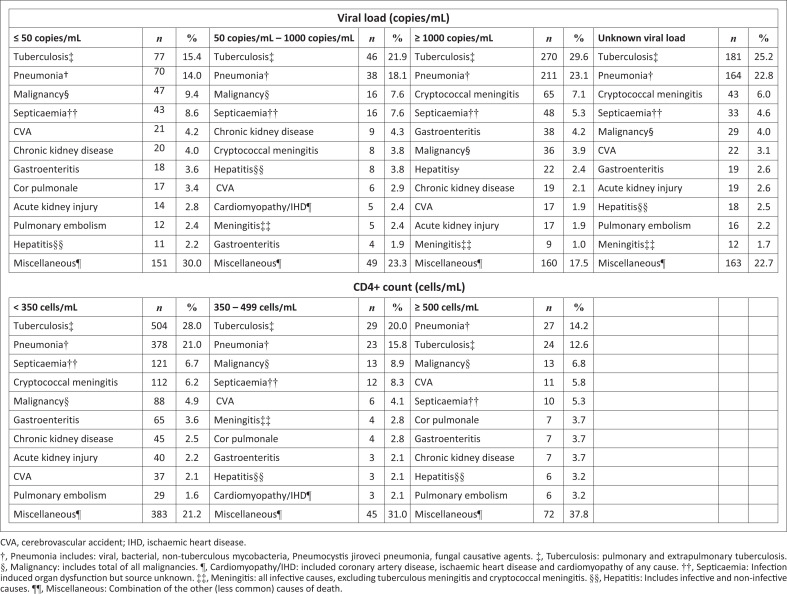
Immediate causes of death in people with HIV, stratified by viral load and CD4+ count.

Infections and parasitic diseases (51%, *n* = 2221) were the leading *immediate* causes of death (Online Appendix 1 Table 2). A large proportion (65%, *n* = 1523) of deaths among PLWH were attributed to infections, even in virally suppressed patients (47.1%, *n* = 236). Communicable diseases were also the leading *immediate* cause of death in HIV-seronegative patients (32.3%, *n* = 318) and those with unknown HIV-serostatus (37.3%, *n* = 380). Viral, bacterial, and fungal pneumonia (19.9%, *n* = 863), pulmonary and extrapulmonary tuberculosis (15.0%, *n* = 654), neoplasms (7.5%, *n* = 325), cardiomyopathy/ischaemic heart disease (6.9%, *n* = 299) and cerebrovascular accidents (6.4%, *n* = 277) were the leading *immediate* causes of death (Table 1, Online Appendix 1 Table 3). Tuberculosis and pneumonia were consistently the leading *immediate* causes of death in PLWH, regardless of the VL and CD4+ count (Figure 4). The specific *immediate* causes of death among HIV-seronegative inpatients included pneumonia (20.5%, *n* = 202) and neoplasms (22.9%, *n* = 127), followed by cardiomyopathy/ischaemic heart disease (10.9%, *n* = 107) and cerebrovascular accidents (7.4%, *n* = 73). The proportions of deaths caused by neoplasms, circulatory, genitourinary and nervous system diseases were higher among virally suppressed patients compared to PLWH overall (Online Appendix 1 Table 2). Similar *immediate* and *underlying* causes of death were noted in HIV-seronegative patients and those with an unknown HIV-serostatus.

**TABLE 1:** Immediate causes of death stratified by HIV-serostatus.

All deaths	*n*	%	HIV-positive	*n*	%	HIV-negative	*n*	%	Unknown HIV status	*n*	%
Pneumonia†	863	19.9	Tuberculosis‡	584	24.9	Pneumonia†	202	20.5	Pneumonia†	178	17.4
Tuberculosis‡	654	15.0	Pneumonia†	483	20.6	Malignancy§	127	12.9	CVA	136	13.3
Malignancy§	325	7.5	Malignancy§	128	5.5	Cardiomyopathy/IHD¶	107	10.9	Cardiomyopathy/IHD¶	128	12.5
Cardiomyopathy/IHD¶	299	6.9	Cryptococcal meningitis	120	5.1	CVA	73	7.4	Malignancy§	70	6.8
CVA	277	6.4	Septicaemia††	115	4.9	Tuberculosis‡	53	5.4	Septicaemia††	62	6.1
Septicaemia††	230	5.2	CVA	68	2.9	Septicaemia††	53	5.4	Aspiration pneumonitis	53	5.2
Cryptococcal meningitis	120	2.8	Cardiomyopathy/IHD¶	64	2.7	Lung cancer	42	4.3	Chronic kidney disease	25	2.5
Aspiration pneumonitis	120	2.8	Gastroenteritis	51	2.2	Chronic kidney disease	42	4.3	COPD	25	2.5
Chronic kidney disease	117	2.7	Acute kidney injury	50	2.1	Aspiration pneumonitis	35	3.6	Pulmonary embolism	19	1.9
Pulmonary embolism	78	1.8	Chronic kidney disease	50	2.1	Pulmonary embolism	18	1.8	Lung cancer	18	1.8
Lung cancer	71	1.6	Kaposi sarcoma	42	1.8	COPD	18	1.8	Tuberculosis‡	17	1.7
Miscellaneous¶¶	1192	27.4	Miscellaneous¶¶	587	25.0	Miscellaneous¶¶	214	21.7	Miscellaneous¶¶	289	28.3

CVA, cerebrovascular accident; IHD, ischaemic heart disease; COPD, Chronic obstructive pulmonary disease.

† , Pneumonia includes: viral, bacterial, non-tuberculous mycobacteria, *Pneumocystis jiroveci* pneumonia, fungal causative agents.

‡ , Tuberculosis: pulmonary and extrapulmonary tuberculosis.

§ , Malignancy: includes total of all malignancies.

¶ , Cardiomyopathy/IHD: included coronary artery disease, ischaemic heart disease and cardiomyopathy of any cause.

†† , Septicaemia: Infection induced organ dysfunction but source unknown.

‡‡ , Meningitis: all infective causes, excluding tuberculous meningitis and cryptococcal meningitis.

§§ , Hepatitis: Includes infective and non-infective causes.

¶¶ , Miscellaneous: Combination of the other (less common) causes of death.

Infectious diseases (46.2%, *n* = 2008) were also the leading *underlying* cause of death overall (Online Appendix 1 Table 2). That largely reflected the high proportion of deaths from communicable diseases among PLWH (82.9%, *n* = 1941). Circulatory (26.9%, *n* = 265) and respiratory (15.6%, *n* = 156) system diseases were the leading *underlying* causes among deaths in hospitalised HIV-seronegative patients. Mental and behavioural disorders, including dementia, occurred more frequently in HIV-seronegative patients (6.4%, *n* = 63) and those with unknown serostatus (5.4%, *n* = 55). However, at the time of death, those categorised as HIV-unknown and/or HIV-seronegative were a median of 21.8 years older (IQR: 6.8–34.4), compared to PLWH. Overall, the leading (specific) *underlying* causes of death in this study were HIV/AIDS (41.7%, *n* = 1487), followed by hypertensive diseases (22.6%, *n* = 806), chronic obstructive pulmonary disease (7.8%, *n* = 278), and septicaemia of undetermined source (6.9%, *n* = 246). Anaemia was reported as a *contributory* cause in 59.5% (*n* = 2585) of all deaths.

After adjusting for age, gender and HIV-serostatus, no differences were noted in the risk of death from pneumonia (Table 2), when comparing patients < 35 years and those aged 35–49 years (aRR: 1.04 [95% CI: 0.85–1.27, *P* = 0.724]). Comparatively, patients above the age of 64 years had an increased risk of dying from pneumonia (aRR: 1.64 [95% CI: 1.36–1.98, *P* < 0.001]). The risk of death from pneumonia was higher in male compared to female inpatients (aRR: 1.13 [95% CI: 1.0–1.28, *P* = 0.043]). No difference was found in the risk of dying from pneumonia among HIV-seronegative patients, when comparing PLWH who had CD4+ counts of ≥ 500 cells/mL (aRR: 1.44 [95% CI: 1.00–2.09, *P* = 0.052]). However, the risk increased when CD4+ counts were < 350 cells/mL (aRR: 1.48 [95% CI: 1.03–2.12, *P* = 0.034]).

**TABLE 2:** Factors associated with risk of death from tuberculosis and pneumonia.

Variables	Inpatient deaths caused by tuberculosis (pulmonary and extrapulmonary)										Inpatient deaths caused by pneumonia (viral, bacterial, fungal)									
	*n*	%	Univariable			*P*	Adjusted			*P*	*n*	%	Univariable			*P*	Adjusted			*P*
			RR	95%	CI		aRR	95%	CI				RR	95%	CI		aRR	95%	CI	
**Age (years)**																				
15 – 34	172	27.7	7.22	5.32	9.80	*	2.14	1.53	2.98	*	119	19.1	1.06	0.87	1.30	0.575	1.04	0.85	1.27	0.724
35 – 49	270	22.6	5.91	4.39	7.95	*	1.80	1.30	2.49	*	217	18.1	1.00	-	-	-	1.00	-	-	-
50 – 64	164	13.0	3.49	2.49	4.66	*	1.61	1.17	2.23	**	238	18.9	1.04	0.88	1.23	0.612	1.20	1.01	1.43	**
≥ 65	48	3.8	1.00	-	-	-	1.00	-	-	-	289	22.9	1.27	1.08	1.49	**	1.64	1.36	1.98	*
**Gender**																				
Female	276	13.6	1.00	-	-	-	1.00	-	-	-	375	18.5	1.00	-	-	-	1.00	-	-	-
Male	378	16.3	1.19	1.03	1.38	**	1.19	1.04	−1.36	*	488	21.0	1.13	1.00	1.28	**	1.13	1.00	1.28	**
**HIV status**																				
HIV-seronegative	53	5.4	1.00	-	-	-	1.00	-	-	-	202	20.5	1.00	-	-	-	1.00	-	-	-
HIV-seropositive	584	24.9	4.63	3.53	6.07	*	1.29	0.77	2.14	0.333	483	20.6	1.00	0.87	1.16	0.951	0.42	0.15	1.15	0.091
HIV-serostatus unknown	17	1.7	0.31	0.18	0.53	*	0.33	0.88	1.42	*	178	17.5	0.85	0.71	1.02	0.079	0.84	0.70	1.01	0.058
**CD4+ count (cells/mL)**																				
HIV-seronegative	53	5.4	0.41	0.26	0.63	*	-	-	-	-	202	20.5	1.44	1.00	2.09	0.052	-	-	-	-
CD4+ count < 350	504	28.0	2.12	1.48	3.04	*	2.10	1.44	3.04	**	378	21.0	1.48	1.03	2.12	**	1.23	0.85	1.80	0.271
CD4+ count 350 – 499	29	20.0	1.52	0.94	2.45	0.087	1.89	1.15	3.11	**	23	15.9	1.12	0.67	1.86	0.674	1.07	0.65	1.77	0.785
CD4+ ≥ 500	24	12.6	1.00	-	-	-	1.00	-	-	-	27	14.2	1.00	-	-	-	1.00	-	-	-
CD4+ count unknown	27	13.2	0.96	0.57	1.60	0.873	1.32	0.77	2.37	0.311	55	26.8	1.89	1.25	2.86	**	1.45	0.93	2.26	0.098
HIV-serostatus unknown	17	1.7	0.13	0.07	0.23	*	-	-	-	-	178	17.5	1.90	0.84	1.79	0.282	-	-	-	-
**Viral load (copies/mL)**																				
HIV-seronegative	53	5.4	0.35	0.25	0.49	*	-	-	-	-	202	20.5	2.46	0.96	6.35	**	-	-	-	-
VL ≤ 50	77	15.4	1.00	-	-	-	1.00	-	-	-	70	14.0	1.68	0.64	4.39	0.293	1.69	0.64	4.45	0.288
VL 51 – 399	33	20.4	1.33	0.92	1.91	0.133	1.12	0.78	1.63	0.534	34	21.0	2.52	0.94	6.74	**	2.56	0.95	6.86	0.062
VL 400 – 1000	13	27.1	1.76	1.06	2.93	**	1.39	0.82	1.63	0.229	4	8.3	1.00	-	-	-	1.00	-	-	-
VL ≥ 1000	270	29.6	1.92	1.53	2.42	*	1.56	1.22	2.00	*	211	23.1	2.78	1.08	7.15	**	2.82	1.09	7.28	**
VL unknown	191	26.6	1.73	1.36	2.19	*	1.61	1.24	2.08	*	164	22.8	2.79	1.06	7.06	**	2.62	1.01	6.79	**
HIV-serostatus unknown	17	1.7	0.11	0.06	0.18	*	-	-	-	-	178	17.5	2.80	0.81	5.40	0.126	-	-	-	-

RR, relative risk; aRR, adjusted relative risk; VL, viral load; CI, confidence interval.

* , *P* < 0.001;

** , *P* ≤ 0.05.

The risk of in-hospital death caused by pulmonary or extrapulmonary tuberculosis was comparatively lower among patients above the age of 64 years (Table 2). Conversely, a two-fold risk of death occurred in patients below 35 years (aRR: 2.14 [95% CI: 1.53–2.98, *P* < 0.001]). Patients aged 35–49 years (aRR: 1.80 [95% CI: 1.17–2.23, *P* = 0.004]), and those aged 50–64 years (aRR: 1.61 [95% CI: 1.17–2.23, *P* = 0.004]) also had higher risks of dying from tuberculosis compared to those 65 years and older. The risk of death was also higher among male compared to female inpatients (aRR: 1.19 [95% CI: 1.04–1.36, *P* < 0.001]).

In univariable analyses, the risks of PLWH dying from pulmonary or extrapulmonary tuberculosis were comparatively higher (aRR: 4.63 [95% CI: 3.53–6.07, *P* < 0.001]). However, after adjusting for covariables, HIV-seropositivity by itself, did not correlate with an elevated risk when compared to HIV-seronegative inpatients (aRR: 1.29 [95% CI: 0.77–2.24, *P* = 0.33]). In PLWH, tuberculosis resulted in a higher risk of death when CD4+ counts were < 500 cells/mL. Risks were up to 2.1-fold when the CD4+ counts were < 350 cells/mL (aRR: 2.10 [95% CI: 1.44–3.04, *P* = 0.009]). Compared to virally suppressed inpatients, those with VL ≥ 1000 copies/mL had a 56% higher risk of in-hospital death from tuberculosis (aRR: 1.56 [95% CI: 1.22–2.00, *P* < 0.001]).

## Discussion

This study showed a decline in overall mortality, at a regional hospital, both for PLWH and those HIV-seronegative or of unknown serostatus. Higher mortality rates persisted in PLWH. Pneumonia and tuberculosis (pulmonary and extrapulmonary) were the leading causes of death, regardless of HIV-serostatus. The risk of dying from pneumonia and tuberculosis was independently associated with age, HIV-seropositivity and, in PLWH, higher VL and lower CD4+ counts.

We postulate that the improved outcomes were likely due to both improved patient care and improved access to ART.^2,3^ Over the study period, Tshepong Hospital improved inpatient HIV testing and counselling, likely leading to earlier HIV diagnosis and ART initiation, improved adherence, and possibly improved overall survival among PLWH. Departmental mortality meetings were used as a learning opportunity to identify shortcomings in patient management, and might have contributed to the improved secular trend in mortality we report.^20,21^

However, our data highlighted the remaining gaps between PLWH and their HIV-seronegative peers. Although PLWH had improved outcomes over time, higher inpatient mortality rates were observed compared to HIV-seronegative inpatients and those with unknown HIV-serostatus.^15,22,23,24,25^ Moreover, deaths in PLWH occurred at younger ages, while HIV-seronegative patients died at ages comparable to those predicted by current life expectancy estimates in South Africa.^8^ The effects of chronic inflammation and premature aging seen in PLWH may represent a possible reason for this discrepancy.^26^ Screening for and treatment of comorbidities at younger ages may be needed for PLWH.^26,27,28^ Initiation of ART closer to the time of HIV infection, improved adherence, and earlier switching of failing regimens may further reduce mortality.^29^

Consistent with other studies in sub-Saharan Africa, the leading cause of death among PLWH was tuberculosis.^15,23,30,31,32,33,34^ Regardless of CD4+ count or VL, PLWH were at an increased risk of dying from tuberculosis compared to HIV-seronegative inpatients. However, risks were reduced in virally suppressed PLWH and those with higher CD4+ counts. The highest risk of death resulting from pneumonia and tuberculosis was observed in men, inpatients with VL ≥ 1000 copies/mL, and CD4+ counts < 350 cells/mL. A concerted effort to improve determinants on a healthcare level, patient education, and to decrease the community transmission of tuberculosis, are needed to address this problem.^30^ The improved mortality outcomes observed in virally suppressed PLWH demonstrates the efficacy of ART in decreasing mortality and improving survival.

In our study, up to 9.3% of reported deaths among PLWH were caused by neoplastic diseases. Similarly, a Zimbabwean study reported increased rates of death resulting from malignancies and non-communicable diseases in PLWH.^23^

South Africa is one of the countries with the highest numbers of HIV/AIDS-related deaths.^35^ We reported HIV and AIDS as the leading specific *underlying* cause of death by a large margin.

A high proportion of inpatients who died reported previous tuberculosis, with disrupted lung architecture frequently seen as a sequela of pulmonary tuberculosis. This may be a possible reason for the high burden of respiratory tract infections observed in this study. When considering all pathological processes, diseases of the respiratory system were involved in 41.5% (*n* = 1801) of all deaths. Collectively, chronic obstructive pulmonary disease and fibro-cavitary lung diseases represented 12.5% of the reported *underlying* causes of death. Strategies promoting smoking cessation, vaccination, and earlier tuberculosis diagnosis may yield further improved outcomes. In South Africa, the first wave of the coronavirus disease 2019 peaked in July 2020.^36^ This coincides with an increased admission and mortality rate from March to June 2020, as some of the admissions and deaths may have been due to coronavirus disease 2019. Anaemia was the leading *contributory* cause of death in our study, reflecting the negative impact of low haemoglobin concentrations on inpatients outcomes.^37,38,39,40^

Furthermore, this study highlights the need to improve inpatient management of communicable diseases. Possible areas that need improvement include earlier recognition and aggressive management of pneumonia and septicaemia, with timely escalation of treatment. Better pneumonia scoring systems, that include HIV infection and CD4+ counts, may also ensure earlier recognition and treatment of high-risk patients.^41^ Further improvement, with linked HIV and tuberculosis care, is needed, together with close communication between inpatient and outpatient care.^30^ Efforts and research into the improving cooperation between the different levels of healthcare, earlier detection of diseases like tuberculosis, as well as education of hospitalised patients may yield further survival benefits. Specific emphasis on using the admission as opportunity to provide healthcare education and counselling on treatment adherence may impact readmissions and patient outcomes.

We evaluated a large data set, lending strength and better internal validity to the findings. However, our study has limitations. The study was conducted at a single centre, limiting external validity. To minimise misclassification, a systematic process was undertaken to review all the deaths that occurred during the period of interest. Misclassification of death is a substantial problem in South African mortality statistics, especially overreporting of tuberculosis as the underlying cause of death instead of using HIV infection.^42,43,44^ Extensive cross-checking of patient records, clinical information, and investigations was performed to classify the causes of death, but the causes of death were not determined by autopsy, which often identifies incorrect premortem diagnoses.^45^ Limited data were available regarding the characteristics of those who survived admission (including final discharge diagnoses), which made it impossible to calculate mortality rates for subgroups stratified by age, VL, and CD4+ count and diagnostic category. Moreover, the duration since HIV diagnosis, the time from diagnosis until treatment initiation, nadir CD4+ counts and other characteristics of patients who died (or survived) their admission, were not available.

## Conclusion

Despite the high burden of communicable diseases, inpatient outcomes have improved. However, PLWH, including those who were virally suppressed, died at younger ages, and experienced a higher burden of communicable diseases compared to HIV-seronegative patients. Higher risk factors for death were seen in male patients, as well as in patients with CD4+ counts < 350 cells/mL and VL > 1000 copies/mL in the 6 months prior to death. As ART coverage increases, and continued improvement is observed in the survival of PLWH, mortality and burden of disease need to be monitored to ensure that trends continue to improve. Future research should be conducted to better understand the causes and trends of inpatient mortality and longevity gap that exists between PLWH and their HIV-seronegative counterparts, and to develop interventions to improve outcomes.

## References

[1] UNAIDS data 2021 | UNAIDS [homepage on the Internet]. [cited 2022 Sep 12]. Available from: https://www.unaids.org/en/resources/documents/2021/2021_unaids_data

[2] Cornell M, Grimsrud A, Fairall L, et al. Temporal changes in programme outcomes among adult patients initiating antiretroviral therapy across South Africa, 2002–2007. AIDS. 2010;24(14):2263–2270. 10.1097/QAD.0b013e32833d45c5 20683318 PMC2948209

[3] Cornell M, Johnson LF, Wood R, et al. Twelve-year mortality in adults initiating antiretroviral therapy in South Africa. J Int AIDS Soc. 2017;20(1):21902. 10.7448/IAS.20.1.21902 28953328 PMC5640314

[4] Otieno G, Whiteside YO, Achia T, et al. Decreased HIV-associated mortality rates during scale-up of antiretroviral therapy, 2011–2016. AIDS. 2019;33(15):2423–2430. 10.1097/QAD.0000000000002374 31764107

[5] Ford N, Boulle A, Egger M. Accounting for and responding to HIV-associated mortality. AIDS. 2016;30(3):521–523. 26765941 10.1097/QAD.0000000000000900

[6] Auld AF, Shiraishi RW, Couto A, et al. A decade of antiretroviral therapy scale-up in Mozambique: Evaluation of outcome trends and new models of service delivery among more than 300,000 patients enrolled during 2004–2013. J Acquir Immune Defic Syndr (1988). 2016;73(2):e11–e22. 10.1097/QAI.0000000000001137 PMC11489885 27454248

[7] Harris TG, Rabkin M, El-Sadr WM. Achieving the fourth 90: Healthy aging for people living with HIV. AIDS. 2018;32(12):1563–1569. 10.1097/QAD.0000000000001870 29762172 PMC6082594

[8] Statistics South Africa. Mid-year population estimates 2022 [homepage on the Internet]. [cited 2022 Sep 13]. Available from: https://www.statssa.gov.za

[9] Rücker SCM, Tayea A, Bitilinyu-Bangoh J, et al. High rates of hypertension, diabetes, elevated low-density lipoprotein cholesterol, and cardiovascular disease risk factors in HIV-infected patients in Malawi. Aids. 2018;32(2):253–260. 10.1097/QAD.0000000000001700 29135581 PMC5757671

[10] Trickey A, May MT, Vehreschild J, et al. Cause-specific mortality in HIV-positive patients who survived ten years after starting antiretroviral therapy. PLoS ONE. 2016;11(8):E0160460. 10.1371/journal.pone.0160460 27525413 PMC4985160

[11] Alejos B, Hernando V, López-Aldeguer J, et al. Overall and cause-specific mortality in HIV-positive subjects compared to the general population. J Int AIDS Soc. 2014;17:19711. 10.7448/IAS.17.4.19711 25397458 PMC4225377

[12] De Coninck Z, Hussain-Alkhateeb L, Bratt G, et al. Non-AIDS mortality is higher among successfully treated people living with HIV compared with matched HIV-negative control persons: A 15-year follow-up cohort study in Sweden. AIDS Patient Care STDS. 2018;32(8):297–305. 10.1089/apc.2018.0015 30067408 PMC6088250

[13] Statistics South Africa. Mortality and causes of death in South Africa, 2016: Findings from death notification [homepage on the Internet]. Statistical South Africa, 2018; p. 1–142. https://www.statssa.gov.za/publications/P03093/P030932016.pdf

[14] Mk Wajanga B, Webster LE, Peck RN, et al. Inpatient mortality of HIV-infected adults in sub-Saharan Africa and possible interventions: A mixed methods review. BMC Health Serv Res. 2014;14:627. 10.1186/s12913-014-0627-9 25465206 PMC4265398

[15] Barak T, Neo DT, Tapela N, et al. HIV-associated morbidity and mortality in a setting of high ART coverage: Prospective surveillance results from a district hospital in Botswana. J Int AIDS Soc. 2019;22(12):e25428. 10.1002/jia2.25428 31850683 PMC6918506

[16] Dawood H, Hassan-Moosa R, Zuma NY, Naidoo K. Mortality and treatment response amongst HIV-infected patients 50 years and older accessing antiretroviral services in South Africa. BMC Infect Dis. 2018;18(1):168. 10.1186/s12879-018-3083-z 29636023 PMC5894176

[17] Matoga MM, Rosenberg NE, Stanley CC, et al. Inpatient mortality rates during an era of increased access to HIV testing and ART: A prospective observational study in Lilongwe, Malawi. PLoS One. 2018;13(2):e0191944. 10.1371/journal.pone.0191944 29415015 PMC5802850

[18] ICD – ICD-10 – International Classification of Diseases, Tenth Revision [homepage on the Internet]. [cited 2020 Jun 20]. Available from: https://www.cdc.gov/nchs/icd/icd10.htm

[19] 2019 ART Clinical Guidelines for the Management of HIV in Adults, Pregnancy, Adolescents, Children, Infants and Neonates. C2019 [cited 2023 Mar 28]. Available from: https://sahivsoc.org/Files/2019%20ART%20Guideline%2028042020%20pdf.pdf

[20] Lau H, Litman KC. Saving lives by studying deaths: Using standardized mortality reviews to improve inpatient safety. Jt Comm J Qual Patient Saf. 2011;37(9):400–408. 10.1016/S1553-7250(11)37050-X 21995256

[21] Long LC, Evans DH, Rosen S, et al. Can routine inpatient mortality data improve HIV mortality estimates? Inpatient mortality at an urban hospital in South Africa. S Afr Med J. 2018;108(10):870–875. 30421717 10.7196/SAMJ.2018.v108i10.13002

[22] Mugisha Okello J, Nash S, Kowal P, et al. Survival of people aged 50 years and older by HIV and HIV treatment status: Findings from three waves of the SAGE-Wellbeing of Older People Study (SAGE-WOPS) in Uganda. AIDS Res Ther. 2020;17(1):17. 10.1186/s12981-020-00276-1 32410634 PMC7226937

[23] Chimbetete C, Shamu T, Roelens M, Bote S, Mudzviti T, Keiser O. Mortality trends and causes of death among HIV positive patients at Newlands Clinic in Harare, Zimbabwe. PLoS One. 2020;15(8):e0237904. 10.1371/journal.pone.0237904 32853215 PMC7451579

[24] Simmons RD, Ciancio BC, Kall MM, Rice BD, Delpech VC. Ten-year mortality trends among persons diagnosed with HIV infection in England and Wales in the era of antiretroviral therapy: AIDS remains a silent killer. HIV Med. 2013;14(10):596–604. 10.1111/hiv.12045 23672663

[25] Burke RM, Henrion MYR, Mallewa J, Masamba L, Kalua T, Khundi M, et al. Incidence of HIV-positive admission and inpatient mortality in Malawi (2012–2019): A population cohort study. AIDS [Internet]. 2021;35(13):2191. 10.1097/QAD.0000000000003006 34172671 PMC7611991

[26] Onen NF, Turner Overton E. A review of premature frailty in HIV-infected persons; Another manifestation of HIV-related accelerated aging. Curr Aging Sci. 2011;4(1):33–41. 10.2174/1874609811104010033 21204781

[27] HIV and Aging | UNAIDS [homepage on the Internet]. [cited 2022 Nov 03]. Available from: https://www.unaids.org/en/resources/documents/2013/20131101_JC2563_hiv-and-aging

[28] Naidoo VA, Martinson NA, Moodley P, et al. HIV prevalence and morbidity in older inpatients in a high HIV prevalence setting. AIDS Res Hum Retroviruses. 2020;36(3):186–192. 10.1089/aid.2019.0137 31631667

[29] Majova B, Variava E, Martinson N. Delays in third-line antiretroviral therapy and outcomes in North West province. South Afr J HIV Med. 2022;23(1):1394. 10.4102/sajhivmed.v23i1.1394 36479420 PMC9634953

[30] Osman M, Karat AS, Khan M, et al. Health system determinants of tuberculosis mortality in South Africa: A causal loop model. BMC Health Serv Res. 2021;21(1):1–11. 10.1186/s12913-021-06398-0 33902565 PMC8074279

[31] Ford N, Matteelli A, Shubber Z, et al. TB as a cause of hospitalization and in-hospital mortality among people living with HIV worldwide: A systematic review and meta-analysis. J Int AIDS Soc. 2016;19(1):20714. 10.7448/IAS.19.1.20714 26765347 PMC4712323

[32] Mishore KM, Hussein N, Huluka SA. Hospitalization and predictors of inpatient mortality among HIV-infected patients in Jimma University Specialized Hospital, Jimma, Ethiopia: Prospective observational study. AIDS Res Treat. 2020;2020:1872358. 10.1155/2020/1872358 32547790 PMC7273427

[33] Colvin M, Dawood S, Kleinschmidt I, Mullick S, Lallo U. Prevalence of HIV and HIV-related diseases in the adult medical wards of a tertiary hospital in Durban, South Africa. Int J STD AIDS. 2001;12(6):386–389. 10.1258/0956462011923336 11368820

[34] Agaba PA, Digin E, Makai R, et al. Clinical characteristics and predictors of mortality in hospitalized HIV-infected Nigerians. J Infect Dev Ctries. 2011;5(05):377–382. 10.3855/jidc.1096 21628815

[35] Gona PN, Gona CM, Ballout S, et al. Burden and changes in HIV/AIDS morbidity and mortality in Southern Africa Development Community Countries, 1990–2017. BMC Public Health. 2020;20(1):1–14. 10.1186/s12889-020-08988-9 32503604 PMC7274054

[36] Jassat W, Mudara C, Ozougwu L, et al. Difference in mortality among individuals admitted to hospital with COVID-19 during the first and second waves in South Africa: A cohort study. Lancet Glob Health. 2021;9(9):e1216–e1225. 10.1101/2021.03.09.21253184 34252381 PMC8270522

[37] Rabe M, Lion-Cachet HC, Eyassu MA. Characteristics and outcomes of older people on antiretroviral therapy in Tlokwe Clinics, South Africa. South Afr J HIV Med. 2020;21(1):1066. 10.4102/sajhivmed.v21i1.1066 32832111 PMC7433252

[38] Meidani M, Rezaei F, Maracy MR, Avijgan M, Tayeri K. Prevalence, severity, and related factors of anemia in HIV/AIDS patients. J. Res. Med. Sci. 2012;17(2):138–142. C2012 [cited 2023 Mar 28]. Available from: https://www.ncbi.nlm.nih.gov/pmc/articles/PMC3525030/pdf/JRMS-17-138.pdf/23264786 PMC3525030

[39] Cao G, Wang Y, Wu Y, Jing W, Liu J, Liu M. Prevalence of anemia among people living with HIV: A systematic review and meta-analysis. EClinicalMedicine. 2022;44:101283. 10.1016/j.eclinm.2022.101283 35128369 PMC8803600

[40] Fonseca C, Araújo M, Moniz P, et al. Prevalence and prognostic impact of anemia and iron deficiency in patients hospitalized in an internal medicine ward: The PRO-IRON study. Eur J Haematol. 2017;99(6):505–513. 10.1111/ejh.12963 28885736

[41] Millman AJ, Greenbaum A, Walaza S, et al. Development of a respiratory severity score for hospitalized adults in a high HIV-prevalence setting-South Africa, 2010–2011. BMC Pulm Med. 2017;17(1):1–8. 10.1186/s12890-017-0368-8 28148246 PMC5288997

[42] Bradshaw D, Msemburi W, Dorrington R, Pillay-Van Wyk V, Laubscher R, Groenewald P. HIV/AIDS in South Africa: How many people died from the disease between 1997 and 2010? AIDS. 2016;30(5):771–778. 10.1097/QAD.0000000000000947 26509830

[43] Dorrington R, Bradshaw D, Laubscher R, Nannan N. Rapid mortality surveillance report. 2018 [homepage on the Internet]. 2020 [cited 2020 Aug 13]. Available from: https://www.mrc.ac.za/bod/reports.htm

[44] Bradshaw D, Pillay-Van Wyk V, Laubscher R, Nojilana B, Groenewald P, Nannan N, et al. Cause of death statistics for South Africa: Challenges and possibilities for improvement. 2010 [cited 2023 Mar 28]. Available from: https://www.samrc.ac.za/sites/default/files/files/2017-05-26/cause_death_statsSA.pdf/

[45] Wong EB, Omar T, Setlhako GJ, et al. Causes of death on antiretroviral therapy: A post-mortem study from South Africa. PLoS One. 2012;7(10):e47542. 10.1371/journal.pone.0047542 23094059 PMC3472995

